# Social anhedonia as a Disrupted-in-Schizophrenia 1-dependent phenotype

**DOI:** 10.1038/s41598-022-14102-3

**Published:** 2022-06-17

**Authors:** Mohammad Seidisarouei, Sandra Schäble, Marijn van Wingerden, Svenja V. Trossbach, Carsten Korth, Tobias Kalenscher

**Affiliations:** 1 Social Rodent Lab, Institute of Experimental Psychology, Heinrich-Heine University, 40225 Düsseldorf, Germany; 2 Comparative Psychology, Institute of Experimental Psychology, Heinrich-Heine University, 40225 Düsseldorf, Germany; 3 Department of Cognitive Science and Artificial Intelligence, Tilburg School of Humanities and Digital Sciences, Tilburg University, Tilburg, The Netherlands; 4 Department of Neuropathology, Medical Faculty, Heinrich-Heine University, Düsseldorf, Germany

**Keywords:** Neuroscience, Psychology

## Abstract

Deficits in social interaction or social cognition are key phenotypes in a variety of chronic mental diseases, yet, their modeling and molecular dissection are only in their infancy. The Disrupted-in-Schizophrenia 1 (DISC1) signaling pathway is considered to play a role in different psychiatric disorders such as schizophrenia, depression, and biopolar disorders. DISC1 is involved in regulating the dopaminergic neurotransmission in, among others, the mesolimbic reward system. A transgenic rat line tgDISC1 has been introduced as a model system to study behavioral phenotypes associated with abnormal DISC1 signaling pathways. Here, we evaluated the impact of impaired DISC1 signaling on social (social interaction) and non-social (sucrose) reward preferences in the tgDISC1 animal model. In a plus-maze setting, rats chose between the opportunity for social interaction with an unfamiliar juvenile conspecific (social reward) or drinking sweet solutions with variable sucrose concentrations (non-social reward). tgDISC1 rats differed from wild-type rats in their social, but not in their non-social reward preferences. Specifically, DISC1 rats showed a lower interest in interaction with the juvenile conspecific, but did not differ from wild-type rats in their preference for higher sucrose concentrations. These results suggest that disruptions of the DISC1 signaling pathway that is associated with altered dopamine transmission in the brain result in selective deficits in social motivation reminiscent of phenotypes seen in neuropsychiatric illness.

## Introduction

Mental diseases such as schizophrenia, depression, and autism spectrum disorder (ASD) are characterized by strongly altered social cognition, the core feature of processing social information^1,2^. For example, impairments in social cognition, manifested as deficits in recognizing emotions, making contact, inferring thoughts, and responding emotionally to others, are seen in all phases of schizophrenia^3^. Although the interrelationship of dysfunction in social cognition and negative symptoms is still open for further discussion^2^, the disrupted social cognitive abilities can be related to withdrawal from social interaction and reduced motivation for engaging in social relationships^4,5^. This reduced motivation for social interaction favors the genesis of co-morbid depression and poor functional or motivational outcomes seen in schizophrenic patients^6,7^. Therefore, given the importance and current masked dimensions of social cognition^2^, testing the established schizophrenic animal models in new paradigms seems crucial.

The Disrupted-in-Schizophrenia 1 (DISC1) protein and its signaling pathway play an important role in mental diseases. The DISC1 gene was originally identified in a Scottish family in which a chromosomal translocation directly disrupts the DISC1 gene, leading to several mental disorders including schizophrenia and recurrent major depression^8,9^. Alterations in the DISC1 gene are associated with impairments in brain development in humans, as well as primates and rodents, explicating a possible mechanism for their role in several psychiatric disorders^10–14^.

Even though no common genetic variants of DISC1 have been reported to be associated with mental illness^15,16^, on a posttranslational level, the DISC1 protein is at the center stage of major signaling pathways relevant for regulating brain functions involved in adaptive behavior^17^. The DISC1 protein's role in neuronal development includes proliferation and migration of the neuronal progenitor cells and synapse formation and maintenance^18^, and it acts as a molecular hub that interacts with dopaminergic neurotransmission components such as Dopamine (DA) D2 receptors and transporter^19–21^. In this regard, the association between DISC1 and the function of DA, one of the leading candidate neurotransmitters in the pathology of different psychiatric disorders, has been profoundly investigated^22–25^. The findings suggest that DISC1 has a role in the dysregulation of DA functions, such as the increase in the proportion of striatal D_2_^high^ receptors^26^, an increase of DAT levels in the striatum^27^, and a decrease of extracellular DA levels in the nucleus accumbens^28,29^.

A novel transgenic rat model (tgDISC1) has been introduced to study the function of the DISC1 protein in disease and normal cognition^21^. The tgDISC1 rat is a model for aberrant DISC1 protein signaling by modestly overexpressing non-mutant human DISC1 leading to DISC1 aggregation and thus representing a subset of sporadic cases with mental illness^30^. Furthermore, studies by different labs^21,31–34^ reported a full signature of behavioral phenotypes that included amphetamine supersensitivity, hyper-exploratory behavior, and rotarod deficits associated with reductions in DA neurotransmission of the tgDISC1 rats. Thus, this tgDISC1 rat model could be exploited to investigate behavioral differences, specifically, variation in reward-related behavior caused by altered dopamine homeostasis.

Anhedonia, a consequence of deficits in reward processing, is one of the core symptoms of psychotic disorders. It is described as a lack of motivational ability in experiencing pleasure and reduced response to rewarding objects such as non-social reward (e.g., food) or social reward (i.e., social interaction)^1,34^. Considering that the DAergic system acts as a leading player in the reward learning process, control of motivation^35^, and encoding the reward prediction error^36,37^, anhedonia might be caused by a dysregulation in DA^38,39^.

In addition to general anhedonia, social interaction and cognition have been linked to DA activity^40,41^, the abnormal social behaviors in mental disease may stem from the pathological reward and DA processes, too^39,42,43^. However, it is unknown whether general, non-social anhedonia and the social deficits seen in psychiatric disorders stem from the same dopaminergic mechanisms or whether they are the consequence of separate, dissociable processes.

This study exploits the tgDISC1 animal model to address whether abnormal DA homeostasis, previously shown in these animals^21^, is linked to reduced non-social reward processing, social interaction seeking, or both. To this end, we compared the choice behavior of tgDISC1 with wild-type rats in a novel paradigm^44^ in which they had to choose between the possibility of social interaction with an unfamiliar juvenile conspecific or drinking sweet solutions with different sucrose concentrations.

## Methods

### Subjects

The experiment was conducted according to the European Union Directive 2010/63/E.U. for animal experimentation, in accordance to all procedure of ARRIVE guideliness and was approved by the local authorities (Landesamt für Natur, Umwelt und Verbraucherschutz North-Rhine Westphalia, Germany). Transgenic DISC1 (tgDISC1) Sprague Dawley rats and their sibling wild-type (WT) littermate controls were bred at the local animal facility (ZETT, Heinrich-Heine University, Düsseldorf, Germany), 36 male Sprague Dawley rats (tgDISC1 = 12, WT = 12, juvenile rats (WT) = 12) in total, consisting of 24 actor rats (PND 57–60, tgDISC1 Mweight = 285 g and WT Mweight = 304 g, at the starting day of the experiment; see supplementary materials 1, Figure S5) and 12 juvenile rats (PND 28, Mweight = 145 g at the starting day of the Social-Sucrose Preference Test (SSPT)), serving as social stimulus rat. Experimental rats were kept in groups of N = 2 for actors and N = 3 for social stimulus rats, in standard Type IV Macrolon cages in a reversed 12:12 h light–dark cycle. The stable room was kept at a constant temperature of 22 °C ± 2 and a humidity of 55% ± 2. Throughout the experiment, all actor rats received standard laboratory rodent food, ad libitum, excepting the Sucrose Discrimination Test (SDT) phase in which all actors were limited in their food intake (food per rat per day: 22 g on weekdays and 25 g on weekends). Notably, group assignments for behavioral testing was randomized within/between-group (tgDISC1 and WT) and within-group for social stimulus rats.

### Screening of transgenic animals

Detection of the transgene was performed as previously described 21. In short, biopsies were digested in a buffer containing 100 mM Tris pH 8,5 mM EDTA, 0.2% SDS, 200 mM NaCl and 100 μg/ml Proteinase K and gDNA precipitated with isopropanol and solubilized in water.

For the quantification of transgene load (heterozygous versus homozygous), quantitative PCR with the StepOnePlus Real-Time PCR System and the Platinum SYBR Green qPCR SuperMix-UDG (both Thermo Fisher Scientific, USA) was performed. Primer transgene: forward 5′-CTGATCTCCAGAAGCCCAAA-3′, reverse 5′-CAGGCCTATTCCTTGACAGC-3′; primer housekeeper beta-actin: 5′-GCAACGCGCAGCCACTGTCG-3′, reverse 5′-CCACGCTCCACCCCTCTAC-3′. Quantitative PCR conditions: 10 min at 95 °C, followed by 40 cycles of 15 s at 95 °C and 60 °C for 1 min. The data were processed with the StepOne Software v2.3 (Thermo Fisher Scientific, USA), and transgene expression was normalized to the expression level of the housekeeper. Only transgenic homozygotes (tgDISC1) and negative littermate control animals (WT) were used for the behavioral studies. Heterozygous and female animals were not included in the study as heterozygous tgDISC1 rats only show subtle phenotypes and a lower gene dose might complicate the interpretation of results. We used only male rats to control for sex and because of co-habitation restriction in the colony room. While we acknowledge that this is a limitation to the study, we therefore excluded female tgDISC1 rats from our study. After screening, the heterozygous and social stimulus rats were euthanized using Carbon Dioxide. Female rats were used for different purposes. All actor rats were euthanized with an overdose of the anaesthetic Pentobarbital.

### Apparatus and behavioral testing

**Figure 1:**
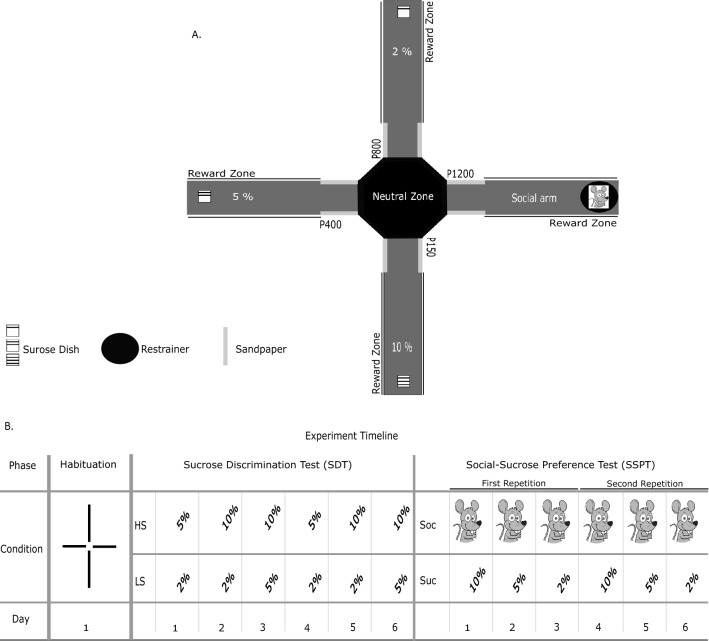
Setup of the study. (**A**) schematic diagram of XCST maze with non-social reward positions, the restrainer for the social reward, and sandpaper positions and grades. (**B**) shows an example of the experiment timeline for different phases, days, and conditions. Habituation: free arm investigation in the habituation phase, Sucrose Discrimination Test (SDT): HS; higher sucrose in a given trial, LS; lower sucrose in a trial, Social-Sucrose Preference Test (SSPT): Soc; social reward, and Suc; sucrose.

Rats were trained in an X-shaped chambered sociability test (XCST). The apparatus was a radial maze (eight-arm), reduced to a cross/plus-maze setup by removing four arms (Fig. 1A), as previously described^44^. The maze consisted of a central octagon zone (36 cm diameter, so-called neutral zone) and four arms (60 cm long and14 cm wide) that extended from the neutral zone. Every arm was consistently associated with one specific reward: 3 arms were assigned to three different concentrations of a sucrose solution reward and one arm with a social stimulus rat (see below for details; Fig. 1A). During all experimental phases, SDT and Social Sucrose Preference Test (SSPT), only 2 out of 4 arms were kept open, depending on the respective task conditions, to provide a direct preference test between two given rewards. One arm was allocated to the social reward (hereinafter as social arm), and an unfamiliar juvenile rat could be placed on this arm in a fixed cylindrical restrainer built from metal bars and compact plastic for its ceiling and floor (Height: 25.5 cm, Diameter: 17 cm, Ugo Basile Sociability Cage). The restrainer was mounted on the maze at the end of the social's arm. The social stimulus rat could move around in the restrainer, and the social/physical contact with the actor rat was possible through the openings between the bars. The sucrose solution was provided to the actor rats in a cube plastic dish (8 × 8 cm) placed at the end of each arm assigned to the non-social reward (i.e., different sucrose concentrations 2%, 5%, and 10%). To facilitate spatial learning of the certain reward in each arm over days, we used sandpaper pieces (17 × 13 cm) attached to the wall at the entrance of each arm that the actor rats would touch with their whiskers when entering the arms. These sandpapers had varying grades (Fig. 1A; 2% sucrose concentration [P800], 5% sucrose concentration [P400], 10% sucrose concentration [P150], and social stimulus [P1200]), following the findings of study^45^, which has shown that rats, through their whiskers, can differentiate between sandpapers with 200 grains/cm^2^ and 25 grains/cm^2^. After each trial, the maze was cleaned by using Ethanol solution (70%).

### Social sucrose preference test

Behavioral testing in the SSPT was divided into three phases: Habituation, SDT, and SSPT (Fig. 1B). In all phases, actor rats from both groups (tgDISC1 & WT) were tested daily. In the first phase (Habituation), all four arms were kept open without rewards. Each actor rat explored the maze for 8 min. This phase intended to determine whether actor rats were inherently biased towards preferring one given arm or sandpaper grade. The second phase was the SDT which was designed to verify that the rats can indeed distinguish among the three selected sucrose levels (2%, 5%, and 10%). To overcome a potential novelty-induced hypophagia, all actor rats were served 40 ml sucrose a day before starting the SDT phase. Food-restricted actor rats were tested on the SDT phase over six days. On each day, they could choose between two sucrose level concentrations in two repetitions of three different conditions (2% vs. 5%, 2% vs. 10%, and 5% vs. 10% in a fixed order, Fig. 1B). In each condition, only two arms were open, and actor rats could freely explore the maze to engage in reward consumption according to their preferences. In each trial, actors were placed in the neutral zone facing not toward open arms at the start of each session (one trial per day). Each trial took 8 min; in this time, actors could drink up to 20 ml sucrose solution per plastic dish mounted at the end of each arm. For each new trial and actor, both dishes were filled with fresh sucrose solution. We estimated the time spent in each arm in each condition (see below). After passing the SDT phase, rats were promoted to the SSPT phase. Before starting the SSPT phase, all social stimulus rats were habituated to the experiment room, maze, and restrainer for three days, each day for 8 min. To keep baseline motivations equal for both types of reward (social & non-social), after the final day of the SDT, food restriction was lifted to let actor rats gain weight over two days before starting the SSPT phase. For the remainder of the experiment, all rats had access to food ad libitum. In the SSPT phase, in each trial with a duration of 8 min, the actor rat could freely explore two open arms: the social arm with the unfamiliar social stimulus rat in the restrainer at the end and one of the arms baited with sucrose at the end. There were three conditions in the SSPT (social reward vs. 2%, social reward vs. 5%, and social reward vs. 10%). As in the SDT phase, actor rats were tested only once per day; the order of conditions was pseudo-randomized across days and rats (Fig. 1B). After completing all conditions, actor rats underwent a second round with the same rat-specific order of conditions as during the first round. Hence, the SSPT phase was completed in six days. Again, we recorded the time spent in each arm on each day of testing as the main index of preference.

In comparison to familiar conspecifics, rats usually spend more time exploring unfamiliar conspecifics^46,47^. Hence, if rats always interact with the same conspecific, the value of social interaction will progressively decline over days with increasing familiarity between rats. To counteract such a trend and maintain the novelty and value of social interaction across testing sessions in the SSPT, twelve different social stimulus rats were used. Thus, each actor rat saw a novel social stimulus rat on each day of testing. The actor-to-social stimulus assignment was counterbalanced across actor rats. We opted for juvenile social stimulus rats, instead of older, adult rats, because a decrease in social interactions and avoidance behavior was previously observed between adult rats^48^. By contrast, juvenile rats show social approach behavior and social play that are assumed to reflect predominantly positive interactions^49,50^.

### Behavioral analysis

Video-Tracking*.* To track the animals' position, we used Ethovision (EthoVision XT version 11.5, Noldus). For each phase of the study (Habituation, SDT, SSPT), different tracking arenas were designed. For the phase of Habituation, each arm was divided into two zones (Sandpaper and Reward zone). For the SDT and SSPT, we used the time that the actor rats spent in the reward zones (Fig. 1A).

### Data analysis

In all analyses, the significance level was set at *p* < 0.05, and all post-hoc tests were Bonferroni-corrected for multiple comparisons. Moreover, the occupancy time for the neutral zone was excluded from all analyses.

### Habituation phase

To test for spatial bias related to any inherent preference for the different reward and sandpaper zones, we performed two separate two-way repeated-measured ANOVA. The first one assessed the effect of group (tgDISC1/ WT) and sandpaper type (four types) as independent variables (IVs) on the time actors spent in each sandpaper zones as dependent variable (DV), and the second one measured the effect of group (tgDISC1/ WT) and reward zones (four zones) as (IVs) on the time actors spent in each reward zones as (DV). This data was collected during habituation to the maze when rewards were not yet introduced.

### Sucrose Discrimination Performance

To determine whether actor rats discriminated between different sucrose levels in the SDT, we calculated the SDT sucrose solution preference score for each condition and repetition in the SDT as a percentage of time spent in the relatively higher sucrose arm (the arm yielding the higher sucrose concentration on that day; Fig. 1B).

$${\text{Higher}}\,{\text{sucrose}}\,{\text{preference}}\,{\text{score}} = \frac{{{\text{Time}}({\text{s}})\,{\text{spent}}\,{\text{in}}\,{\text{higher}}\,{\text{sucrose}}\,{\text{zone}}}}{{({\text{Time}}({\text{s}})\,{\text{spent}}\,{\text{in}}\,{\text{higher}}\,{\text{sucrose}}\,{\text{zone}} + {\text{Time}} ({\text{s}})\,{\text{spent}}\,{\text{in}}\,{\text{lower}}\,{\text{sucrose}}\,{\text{zone}})}}*100$$

By using these sucrose preference scores, we conducted a three-way mixed ANOVA on the higher sucrose zones preference score (DV) with the group as a between-subject factor (tgDISC1 vs. WT), condition (three levels: *2% vs. 5%, 2% vs. 10%,* and *5% vs. 10%)* and test repetition (first vs. second) as within-subject factors.

To control for potential differences in motor activity between tgDISC1 and WT rats, we additionally measured the distance moved (in cm) per day in the entire maze. We ran an independent samples t-test to analyse the group (tgDISC1/ WT) effect on the distance moved (included all repetitions and conditions).

In addition, to determine each group's preference for a higher or lower sucrose reward in a given SDT condition, we also conducted six one-sample t-tests against indifference (50%) on the SDT preference values for higher sucrose at the second repetition.

To explore whether there was a between-group difference in the correlation between *Entrance frequency* and total *duration of stay* **(**in seconds) in a reward zone, we ran two separate Pearson correlations per group: (1) in the higher sucrose zone. (2) in the lower sucrose zone on the mean respective value of each actor rat across all conditions and repetitions.

### Social-Sucrose Preference Analysis

For the SSPT, we calculated a social reward preference score:

$${\text{SSPT}}\,{\text{social}}\,{\text{reward}}\,{\text{preference}}\,{\text{score}} = \frac{{{\text{Time}} ({\text{s}})\,{\text{in}}\,{\text{the}}\,{\text{social}}\,{\text{reward}}\,{\text{zone}}}}{{({\text{Time}}({\text{s}})\,{\text{in}}\,{\text{the}}\,{\text{social}}\,{\text{reward}}\,{\text{zone}} + {\text{Time}}({\text{s}})\,{\text{in}}\,{\text{the}}\,{\text{sucrose}}\,{\text{zone}})}}*100$$

To analyse between-group differences in preference between social (social stimulus rat) or non-social rewards (different sucrose concentrations), we ran a three-way mixed ANOVA to assess the effect of group (tgDISC1 vs. WT), repetition (first vs. second), and condition (*social reward vs. 2%, social reward vs. 5%, social reward vs. 10%*) on the social reward preference score.

Additionally, to determine each group's preference for social or non-social reward in a given SSPT condition, we conducted six one-sample t-tests versus indifference (50%) on the SSPT social reward reference scores, averaged per animal across the two experimental repetitions.

Again, to determine if there was a difference in the distance moved (cm) between tgDISC1 and WT actors, we conducted an independent samples t-test.

Similar to the SDT, we explored whether there was a between-group difference in the correlation between *Entrance frequency* and total *duration of stay* (in seconds) in a reward zone. We ran two separate Pearson correlations per group: (1) in the social reward zone. (2) in the sucrose zone on the mean respective value of each actor rat across all conditions and repetitions.

### Software

All statistical analyses were carried out using SPSS Statistics (version 24; IBM, USA), and figures were created using Jupyter Notebook^51^ through the packages matplotlib^52^, pandas^53^, ptitprince^54^ and seaborn^55^. For improvement of figures, we used Inkscape^56^.

## Results

### Habituation phase

To investigate a potential spatial bias related to any inherent preference for the different reward zones and sandpapers, we executed two distinct repeated measures ANOVAs. These analyses showed no significant bias for either the sandpaper identity or a spatial reward zones location (Table 1).

**Table 1:** Assessment of inherent bias toward a sandpaper or a reward zone.

	Sandpaper zone				Reward zone			
	df	f	*p* value	n^2^_p_	df	f	*p* value	n^2^_p_
Group	1	.105	.752	.009	1	.034	.857	.003
Zone	1.81	2.34	.125	.176	1.67	2.81	.093	.204
Group*Zone	1.67	.387	.648	.034	3	.483	.697	.042

### Sucrose discrimination test

**Figure 2:**
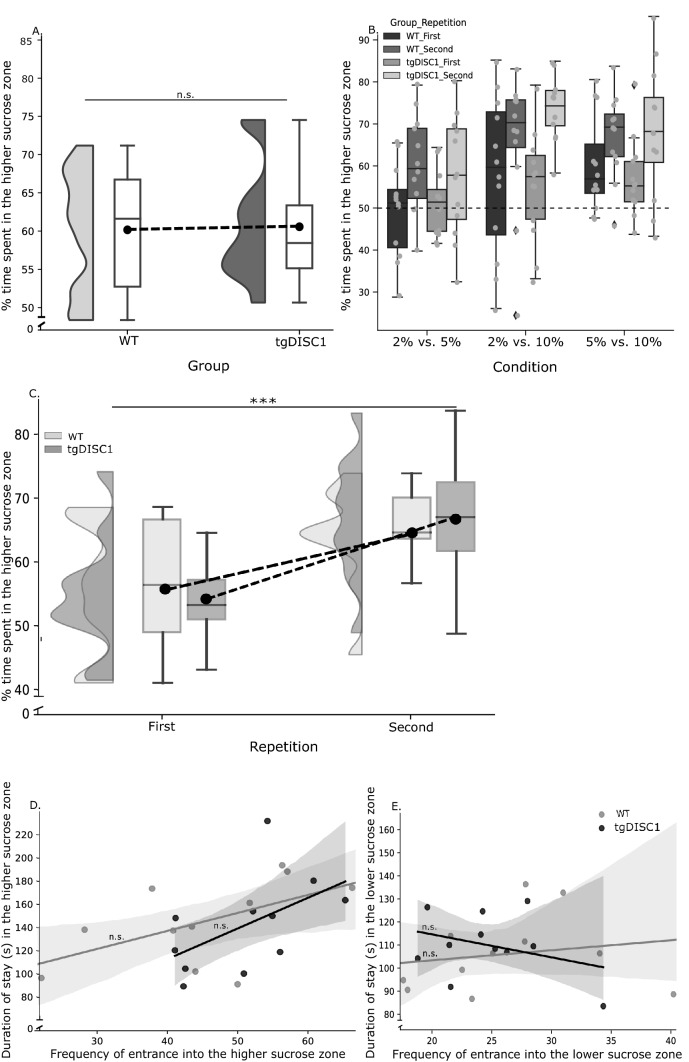
Sucrose Discrimination Test Results. (**A**) The time spent in higher sucrose zones per group included all conditions and repetitions of SDT. The raincloud and whisker plots show the between-group differences in the distribution of time spent in the higher sucrose zone per group. The dashed line connects each group's mean of time spent (across all conditions and repetitions) in the higher sucrose zone. (**B**) Time spent in the higher reward zone in each condition; The dashed line indicates the indifference point (50%). (**C**) The change of time spent in the higher sucrose, per repetition and group. The raincloud and whisker plots show the change in the distribution of time spent in the higher sucrose zone per repetition and group. The dashed line connects each group's mean of time spent in the higher sucrose zone per repetition. (**D**) Correlation between duration of stay and frequency of entrance in higher sucrose zone. Each data point represents the mean across all conditions and repetitions for one actor rat. The gray zones represent standard error. (**E**) Correlation between duration of stay and frequency of entrance in lower sucrose zone. Each data point represents the mean across all conditions and repetitions for one actor rat. The gray zones represent standard error. Error bars represent the standard error of the mean (SEM). ***p < .001, n.s.; not significant. In whisker plots, error bars represent the minimum and maximum of data sets.

To determine whether actor rats could discriminate between different reward sucrose concentrations (2%, 5%, and 10%), we conducted a three-way mixed ANOVA. Our results did *not* show a significant main effect of group (tgDISC1 vs. WT) on the time spent in the respective higher reward zone (F (1,22) = 0.001, *p* = 0.978, ANOVA, Fig. 2A), but we did find a main effect of condition (2% vs. 5% < 2% vs. 10 and 5% vs. 10%; F(1.57,34.5) = 13.7, *p* ≤ 0.001, ANOVA, Fig. 2B) and repetition (F(1,22) = 29.4, *p* ≤ 0.001, ANOVA, Fig. 2C). Bonferroni-corrected post hoc tests revealed that all rats spent more time in the relatively higher sucrose zone in all three conditions; they spent more time in the 10% zone when the alternative was 5% or 2% sucrose, and they spent more time in the 5% than the 2% zone (Table 2A; Fig. 2B). This suggests that all rats were sensitive to relative differences in sucrose concentrations. The Bonferroni test (Table 2B) also showed that rats in both groups significantly spent more time in the higher sucrose zones on the second compared to the first repetition, reflecting learning of the spatial reward arrangement (Fig. 2C). The analysis did not reveal any significant interaction effect (Table 3A). Overall, these results demonstrate that all rats learned to express a clear preference order from high to low sucrose. It has been suggested that the dysregulation in dopaminergic signaling in tgDISC1 rats goes along with locomotor hyperactivity^21^. Hence, to ensure that there was no systematic difference in locomotion between tgDISC1 and WT rats in our tasks, we compared the total distance moved across all conditions and repetitions between animal groups. However, we found no significant difference in distance moved between tgDISC1 and WT rats ((t (22) = 0.101, *p* = 0.921, (tgDISC; [M = 3536, SE = 125] WT; [M = 3516, SE = 148]), for more details, see Table 2F, G). The post-hoc one-sample t-tests against indifference (50%) revealed that all rats spent more time in the higher sucrose zone than in the lower sucrose zone in all conditions, at the second repetition (see supplementary materials, Table S1). Finally, we computed two Pearson correlations per group to determine whether there was a significant correlation between the *frequency of entrance* and *duration of stay* in each zone. The results did not reveal any significant correlations (Table 2C, Fig. 2D,E). For more information about between-group differences in the duration of stay in the higher and lower sucrose zones, see (supplementary materials, figure S4. A and B).

**Table 2:** The results of the pairwise comparisons (Bonferroni) for within-subject factors (Condition/Repetition) in both tasks (SDT/SSPT) on IVs (Time in reward zone/Distance moved) respectively and the result of Pearson correlations in both tasks (SDT/SSPT).

A	Condition	Mean(%)	Sd.Error	Conditions	*p* value	Group Mean (%)	Group-Std.Error
SDT: Time in higher sucrose zone	2% vs 5%	54.5	1.76	2% vs 10%	.001	WT = 54.8	WT = 2.54
				5% vs 10%	.000	tgDISC1 = 54.2	tgDISC1 = 2.4
	2% vs 10%	63.7	2.59	2% vs 5%	.001	WT = 62.7	WT = 3.63
				5% vs 10%	1.000	tgDISC1 = 64.7	tgDISC1 = 2.8
	5% vs 10%	63.2	1.98	2% vs 5%	.000	WT = 63.7	WT = 2.26
				2% vs 10%	1.000	tgDISC1 = 62.7	tgDISC1 = 2.8

B	Repetition	Mean(%)	Sd.Error	Repetition	*p* value	Group Mean(%)	Group-Std.Error
SDT: Time in higher sucrose zone	First	55.4	2.04	Second	.000	WT = 56.2	WT = 2.4
						tgDISC1 = 54.5	tgDISC1 = 1.7
	Second	65.6	2.04			WT = 64.6	WT = 2.15
						tgDISC1 = 66.5	tgDISC1 = 2.4

C	Group	Zone					
Correlation: between duration of stay and frequency of entrance in SDT	WT	Higher	Lower				
		Pearson’r. 557 *p* value .075	Pearson’r. 557 *p* value .595				
	tgDisc1	Pearson’r. 532 *p* value .092	Pearson’r. 321 *p* value .335				

D	Condition	Condition-Mean(%)	Condition-Sd.Error	Conditions	*p* value	Group Mean(%)	Group-Std.Error
SSPT: Time in social reward zone	Social reward vs. 2%	62.5	1.27	Social reward vs. 5%	1.000	WT = 66.2	WT = 1.26
				Social reward vs. 10%	.135	tgDISC1 = 58.9	tgDISC1 = 1.9
	Social reward vs. 5%	61.9	1.4	Social reward vs. 2%	1.000	WT = 66.0	WT = 1.9
				Social reward vs. 10%	.116	tgDISC1 = 57.8	tgDISC1 = 1.9
	Social reward vs. 10%	57.3	2.1	Social reward vs. 2%	.135	WT = 58.7	WT = 3.0
				Social reward vs. 5%	.116	tgDISC1 = 55.9	tgDISC1 = 1.7

E	Repetition	Mean(%)	Sd.Error	Repetition	*p* value	Group Mean(%)	Group-Std.Error
SSPT: Time in social reward zone	First	62.4	1.17	Second	.017	WT = 64.3	WT = 1.5
						tgDISC1 = 60.6	tgDISC1 = 1.7
	Second	58.7	1.48			WT = 63.0	WT = 2.1
						tgDISC1 = 54.4	tgDISC1 = 1.1

F	Condition	Condition-Mean(cm)	Condition-Sd.Error	Conditions	*p* value	Group Mean (cm)	Group-Std.Error
SDT: distance moved	2% vs 5%	3855	108	2% vs 10%	.059	WT = 3767	WT = 140
				5% vs 10%	.000	tgDISC1 = 3943	tgDISC1 = 138
	2% vs 10%	3503	108	2% vs 5%	.059	WT = 3551	WT = 169
				5% vs 10%	.232	tgDISC1 = 3455	tgDISC1 = 144
	5% vs 10%	3275	123	2% vs 5%	.000	WT = 3333	WT = 180
				2% vs 10%	.232	tgDISC1 = 3216	tgDISC1 = 129

G	Repetition	Mean(cm)	Sd.Error	Repetition	*p* value	Group Mean(cm)	Group-Std.Error
SDT: distance moved	First	3676	108	Second	.035	WT = 3537	WT = 94
						tgDISC1 = 3814	tgDISC1 = 91
	Second	3412	105			WT = 3563	WT = 214
						tgDISC1 = 3262	tgDISC1 = 133

H	Condition	Mean(cm)	Sd.Error	Conditions	*p* value	Group Mean (cm)	Group-Std.Error
SSPT: distance moved	Social reward vs. 2%	5433	293	Social reward vs. 5%	.008	WT = 5364	WT = 305
				Social reward vs. 10%	.051	tgDISC1 = 5502	tgDISC1 = 316
	Social reward vs. 5%	6645	334	Social reward vs. 2%	.008	WT = 6648	WT = 485
				Social reward vs. 10%	1.000	tgDISC1 = 6641	tgDISC1 = 390
	Social reward vs. 10%	6541	276	Social reward vs. 2%	.051	WT = 6263	WT = 309
				Social reward vs. 5%	1.000	tgDISC1 = 6109	tgDISC1 = 286

I	Repetition	Mean(cm)	Sd.Error	Repetition	*p* value	Group Mean(cm)	Group-Std.Error
SSPT: distance moved	First	6040	175	Second	.065	WT = 5658	WT = 171
						tgDISC1 = 6421	tgDISC1 = 289
	Second	6373	277			WT = 6525	WT = 404
						tgDISC1 = 6423	tgDISC1 = 292

J	Group	Zone					
Correlation: between duration of stay and frequency of entrance in SSPT	WT	Social reward	Sucrose				
		Pearson’r. 951 *p* value .000	Pearson’r. 573 *p* value .052				
	tgDisc1	Pearson’r. 432 *p* value .335	Pearson’r. 334 *p* value .289

**Table 3:** The results of three-way mixed ANOVA (not-significant interaction effects) in both tasks (SDT/SSPT).

SDT phase (Time in higher sucrose zone)	f-value	*p* value	n_2_^p^
**A**			
Group*Repetition	(1,22) = .956	.339	.042
Group*condition	(2,44) = .711	.344	.015
Condition*Repetition	(2,44) = .577	.566	.026
Group*condition*Repetition	(2,44) = 1.83	.175	.076

SSPT phase (Time in social reward zone)	f-value	*p* value	n_2_^p^
**B**			
Group*Repetition	(1,22) = 2.88	.104	.116
Group*condition	(2,44) = .956	.392	.042
Condition*Repetition	(2,44) = .063	.906	.003
Group*condition*Repetition (2,44) = 1.09	.344	.047	

### Social sucrose preference test

**Figure 3:**
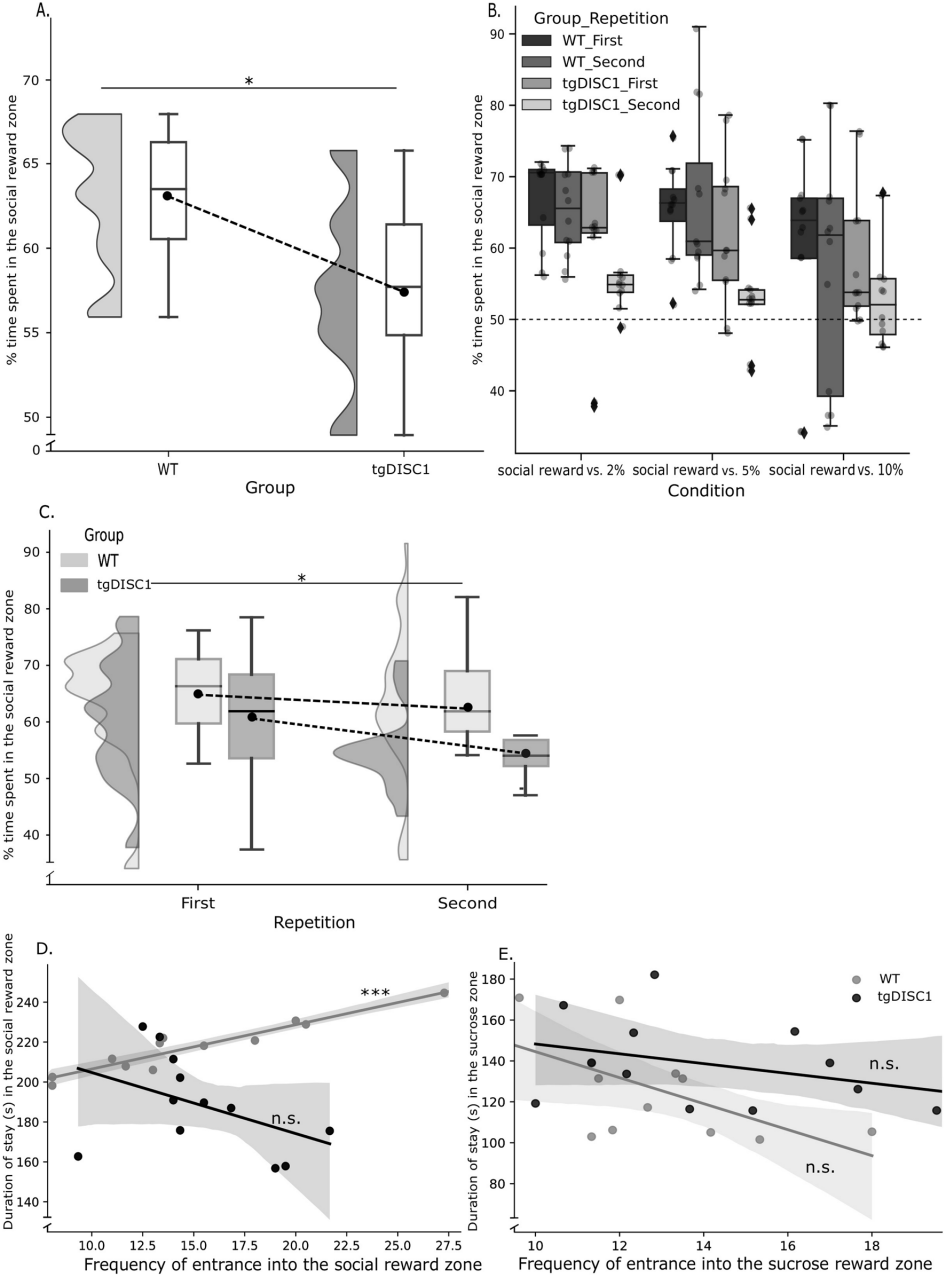
Social Sucrose Preferences Results. (**A**) The time spent in the social reward zone per group included all conditions and repetitions of SSPT. The raincloud and whisker plots show the between-group differences in the distribution of time spent in the social reward zone per group. The dashed line connects each group's mean of time spent (including all conditions and repetitions) in the social reward zone. (**B**) Time spent in the social reward zone. The dashed line indicates the indifference point (50%). (**C**) The change of time spent in the social reward zone per repetition and group. The raincloud and whisker plots show the change in the distribution of time spent in the social reward zone per repetition and group. The dashed line connects each group's mean of time spent in the social reward zone per repetition. (**D**) Correlation between duration of stay and frequency of entrance in social reward zone, per group. Each data point represents the mean of all conditions and repetitions for one actor rat. The gray zones represent standard error (**E**) Correlation between duration of stay and frequency of entrance in sucrose zone, per group. Each data point represents the mean of all conditions and repetitions for one actor rat. The gray zones represent standard error. Error bars represent the SEM. *p < .05, **p < .01, ***p < .001, n.s.; not significant. In whisker plots, error bars represent the minimum and maximum of data set.

To investigate between-group differences in the times spent in the respective rewards zones in the SSPT, we ran a three-way mixed ANOVA on the social reward preference score. This analysis revealed a significant difference between groups in the social reward preference score (F (1,22) = 7.3, *p* = 0.013, ANOVA, Fig. 3A). This result showed that tgDISC1 rats spent a significantly shorter proportion of time (M = 57.5%, SE = 1.0) in the social reward zone than the WT rats (M = 63.6%, SE = 1.3). There was also a significant main effect of sucrose on the time spent in the social reward zone (F (2,44) = 3.7, *p* = 0.032, ANOVA, Fig. 3B). Descriptively, WT rats spent more time in the social reward zone when the alternative was a 2% or a 5% sucrose solution than when the alternative was a 10% solution. However, Bonferroni adjusted post-hoc comparisons (Table 2D) did *not* reveal a significant difference in the percent time rats spent in the social reward zone between the conditions. Accordingly, post-hoc one-sample t-tests against indifference (50%) revealed that all rats spent more time in the social reward zone than in the sucrose zone in all conditions (see supplementary materials, Table S2). Next, we also found a significant main effect of repetition on the proportion of time spent in the social reward zone (F (1,22) = 6.6, *p* = 0.017, ANOVA, Fig. 3C). Rats spent significantly more time in the social reward zone on the first than on the second repetition (Table 2E). There were no significant interaction effects (Table 3B). To make sure that the difference in preference for the social zone between tgDISC1 and WT rats was not the consequence of a general difference in locomotor activity^21^, we, again, compared the total distance moved across all conditions and repetitions between animal groups in the SSPT. However, as in the SDT, we found no significant difference in distance moved between tgDISC1 and WT rats ((t (22) = 0.793, *p* = 0.436, (tgDISC; [M = 6372, SE = 297] WT; [M = 6041, SE = 293], for more details, see Table 2H, I), suggesting that the tgDISC1 effects on social preference are unlikely the result of altered locomotion behavior. Finally, we ran two Pearson correlations per group to determine whether there was a significant correlation between the *frequency of entrance* and *duration of stay* in each zone (sucrose and social rewards). We found a significant correlation between those variables (Table 2J) in the WT rats in the social rewards zone (r (12) = 0.954, *p* ≤ 0.001, Fig. 3D), indicating that those WT rats that entered the social zone more often also stayed longer. This relationship could not be found in the tgDISC1 rats in the social rewards zone (r (12) = -0.432, *p* = 0.161, see Table 2J and Fig. 3E). For more information about between-group differences in the duration of stay in the social and sucrose reward zones, see (supplementary materials, Figure S4. C&D) Taken together, all rats showed a preference for the social over the sucrose reward in all conditions, but the preference strength, indicated by the percent time interacting with the juvenile, was higher in the WT than the tgDISC1 rats.

## Discussion

In this study, we evaluated the effect of aberrant DISC1 signaling on rat behaviour in a paradigm relevant to social cognitive deficits in mental disease: rats could choose between two types of rewards: sweet solutions at variable sucrose concentrations (non-social reward) and the opportunity to interact with a juvenile conspecific (social reward). In our sucrose discrimination task, we found that WT as well as tgDISC1 rats successfully distinguished between sucrose levels and revealed a clear, well-structured preference for higher sucrose concentrations. Hence, we found no evidence to assume an effect of aberrant DISC1 signaling on basic, non-social reward processing. However, when given the choice between drinking sucrose solution or interacting with a conspecific, tgDISC1 rats spent less time with the conspecific than the WT rats, but more time in the non-social reward zones. This might either suggest that, compared to WT rats, tgDISC1 rats had reduced interest in social contact, or that they were lured away from the social interaction zone by the prospect of ingesting more sugar solution in the sucrose zones. However, we consider the latter explanation unlikely since, in the SDT, we found no difference in sucrose preference and sucrose reward-seeking behavior between tgDISC1 and WT rats, suggesting that the reduced time that tgDISC1 rats spent with the conspecific in the SSPT was probably not due to hypersensitivity to sucrose rewards, but the result of genuinely reduced interest in social contact.

By what mechanisms could tgDISC1 rats attach less value to social interaction? It is plausible to assume that this was the result of altered DA signaling in the brain, in particular in the mesolimbic reward system. The reported decreased basal level of DA in striatal samples of tgDISC1 rats was caused by increased D2 receptor and striatal dopamine transporter (DAT) levels, resulting in much faster synaptic DA clearance due to an upregulation of presynaptic DAT^21^. Notably, the upregulation of presynaptic DAT leads to lower net synaptic DA in tgDISC1s. In mice^57^, it is shown that an oxytocin-dependent DAergic projection from the VTA to the NAcc Shell region is necessary and sufficient to support real-time social conditioned place preference, strongly suggesting that an altered DA turnover in the NAcc could interfere with social interaction preferences.

Developmentally, in terms of neuro development, the previous results on the modification (increase in binding) of D2 receptor density by the change in the social environment (social isolation)^58^, the role of DAT levels in the regulation of social behaviors (by DAT knockout of mice)^59^, and the highlighted interplay of regular social contact and striatal function^60^, all suggest that striatal DA signaling is critical for proper social interactions. Behaviorally, in terms of behavior, the tgDISC1s rats' reduced motivation to seek out juvenile conspecifics interaction opportunities aligns with previous studies with neuropsychiatric patients who revealed similar dissociations between social and non-social reward processing^61–63^. For example, patients with schizophrenia may experience impairment and disconnection between several components of social motivation required for interactions with positive social outcomes. Likewise, the result of an investigation^64,65^ found the selective anhedonia (diminished enjoyment) only for social and not non-social reward in children with ASD. A more recent study^64^ reported a decreased reward prediction error signaling (a critical component of reward-based learning) in frontal brain regions only for social reward in patients with ASD, in line with insensitivity to social rewards found for this group^66^.

Another study^67^ also reported a finding pointing to the distinctiveness of social and non-social information processing in schizophrenia and suggested that individuals with schizophrenia may show a selective impairment in processing social stimuli. Likewise, in depression, an association between elevated depressive symptoms and decreased approach to social reward (social feedback) was reported; however, in the same study, the results showed a higher effort by individuals with elevated depression for food rewards^68^.

In summary, our results demonstrate that the tgDISC1 rat features deficits in social interaction and thus is a possible model for this phenotype relevant in schizophrenia or other mental diseases. The here presented social deficits of the tgDISC1 rat align well with the goals of the Measurement and Treatment Research to Improve Cognition in Schizophrenia (MATRICS) that has included social cognition as a major category for biology-based monitoring of clinical trials^69^.

### Limitations and future directions

Rats use different sensory inputs (auditory, olfactory, and visual) in their social interactions. However, it is thought that the most significant rewarding aspect of social interactions for rats is thigmotactic stimulation. In addition, providing a sufficiently large spatial area for social interaction plays an important role in reward experience as well^70,71^. In our design, however, rats could only interact through steel bars which potentially decreases the subjectively rewarding experience of the social interactions. Therefore, in future studies, improving the design in a way that facilitates social interactions is recommended.

Furthermore, we did not test the olfactory performance of the animals, which is a key factor in social behavior^72^. Considering that dopaminergic transmission plays an important role in the olfactory circuit^73^, it might be informative to investigate the difference of olfactory performance between tgDISC1 and WT in future studies.

It has been pointed out that despite activation of the same brain area (ventromedial prefrontal cortex) by both types of rewards (social/non-social), certain areas, such as the amygdala, are more specifically involved in social reward and social cognition^69^. Alongside other study^74^, and we recently demonstrated that amygdala lesions reduce prosocial behavior in rats^75^. This possible regional specificity^76^ might open up possibilities for local DA transmission reinstatement with the aim of rescuing the DISC1 impairment in social reward processing shown here.

In addition, to design this study we relied on the neuronal findings of study^21^ which was performed only with male tgDISC1 rats, thus, we did not use female rats, which should also be considered in future studies.

Last but not least, rats communicate through ultrasonic vocalizations (USV) by employing certain call types that are tuned towards social and non-social conditions^44^. Therefore, in future studies, in addition to the neuronal investigations, recording and analysing USVs in a similar design could shed more light on differences in the subjective affective state of the rats.

Taken together, the results of this study align with previously found associations between DISC1 and neuronal/behavioural impairments and differences in social vs. non-social reward processing in patients with various psychiatric disorders, suggesting that the tgDISC1 animal model is sensitive to capture the altered social reward processing seen in psychiatric illness, qualifying it as a potential standard for understanding the neural and psychopharmacological basis of abnormal social behavior in mental diseases.

## Supplementary Information

Supplementary Information.

## Data Availability

The datasets collected and analysed for this study are available from the corresponding author on reasonable request.
