# Supplementary material for: Social anhedonia as a Disrupted-in-Schizophrenia 1-dependent phenotype
Source: Sci Rep. 2022 Jun 17;12:10182. doi: 10.1038/s41598-022-14102-3 (PMC9205858; doi:10.1038/s41598-022-14102-3)
Supplement: Supplementary file 1 — Supplementary Information. [file 41598_2022_14102_MOESM1_ESM.docx]

Supplemental Material

# Supplemental Results

The social stimulus rats were placed in a restrainer, which potentially induced stress. It is possible that the tgDISC1 rats responded more strongly to the social stimulus rats’ distress signals than WT rats, and, thus, avoided the social reward zones.

To address this issue, we recorded ultrasonic vocalizations (USV) of rats during this experiment. Many studies have shown that rats vocalize 22 kHz USVs in stressed, anxious, and fearful states^1–3^. 22 kHz vocalization calls are, thus, a good indicator of the stress level of the social stimulus rats. If the tgDISC1 rats were indeed more sensitive to the social stimulus rats’ stress levels, we would expect the frequency of 22 kHz USV calls to correlate with the tgDISC1 choice behavior, but less so with the WT rats’ choice behavior. However, we did not find a significant correlation between the frequency of 22 kHz USV calls and the time spent in the social reward zone (see Fig. S1).

In addition, we see the largest difference in minute-by-minute social interaction in the later minutes of a trial (Fig. S2), which is, however, when the number of 22 kHz USVs was lowest (end of the first repetition, see Fig. S3). If the tgDISC1 animals were more negatively impacted by the social stimulus rats’ 22 kHz USVs, one would expect they would match the WT animals in this period. But this is not what we see; instead, we see a sustained diminished choice for social interaction (Fig. S2).

Together, this suggests that the difference in social choice behavior between tgDISC1 and WT rats is unlikely due to a difference in sensitivity to the social stimulus rats’ distress calls but more likely because of an inherent difference in social hedonia.


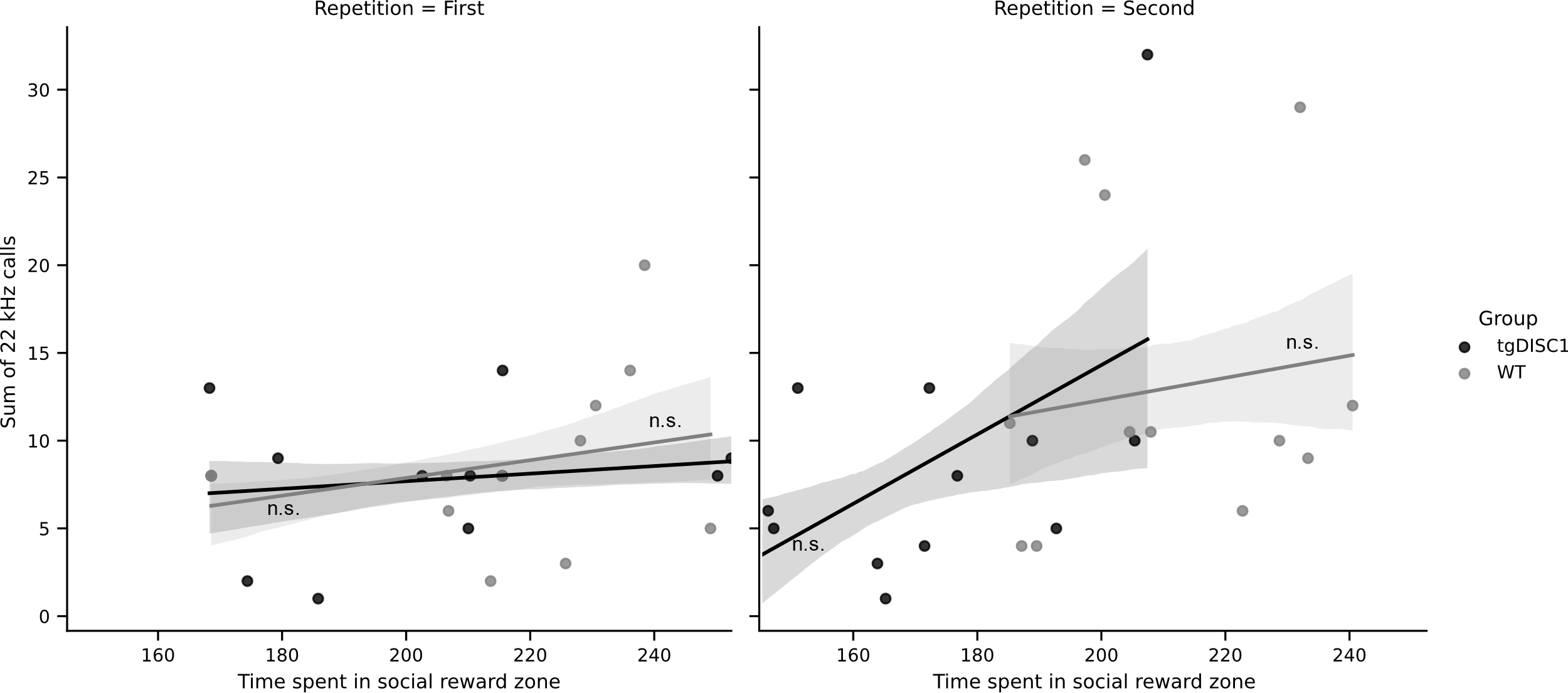


**Figure S1.** Correlation of the time (s) spent in the social reward zone and the number of 22 kHz USVs per group across two repetitions in the social reward zone. Each data point represents the mean of each actor rat across all conditions. n.s.: not significant**.**

**
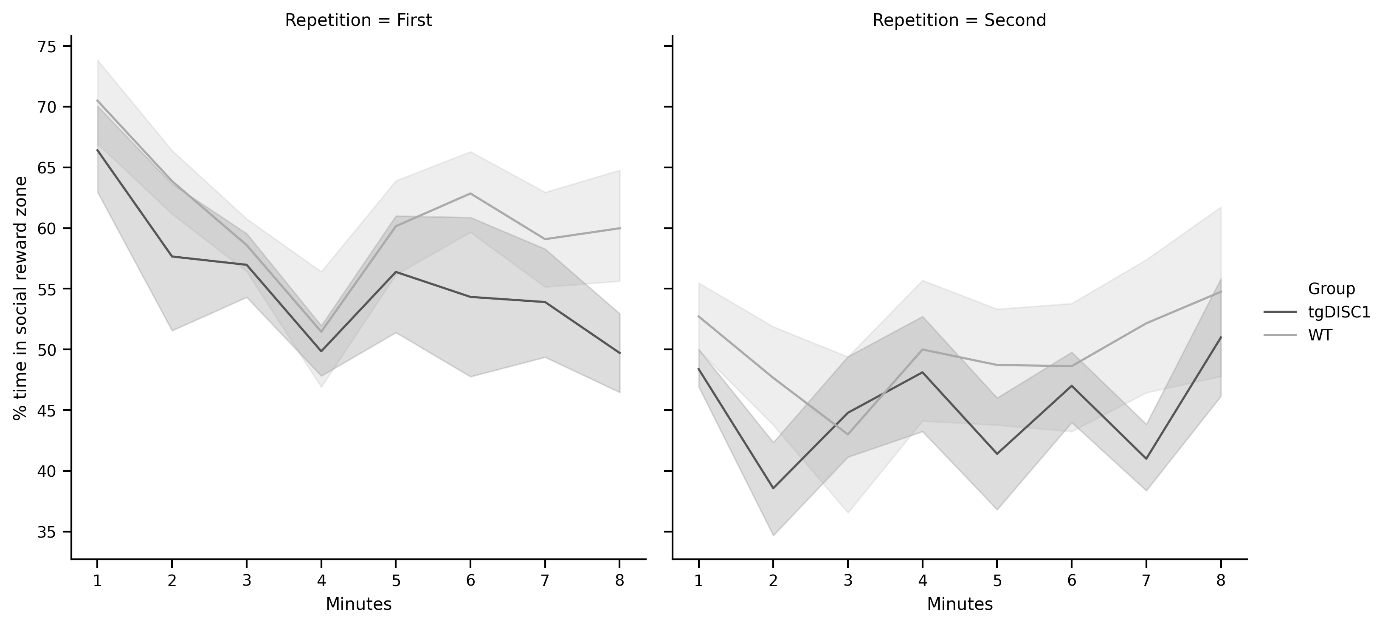
**

**Figure S2**. The groups’ mean per minute across all conditions of each repetition in % of time spent in the social reward zone.


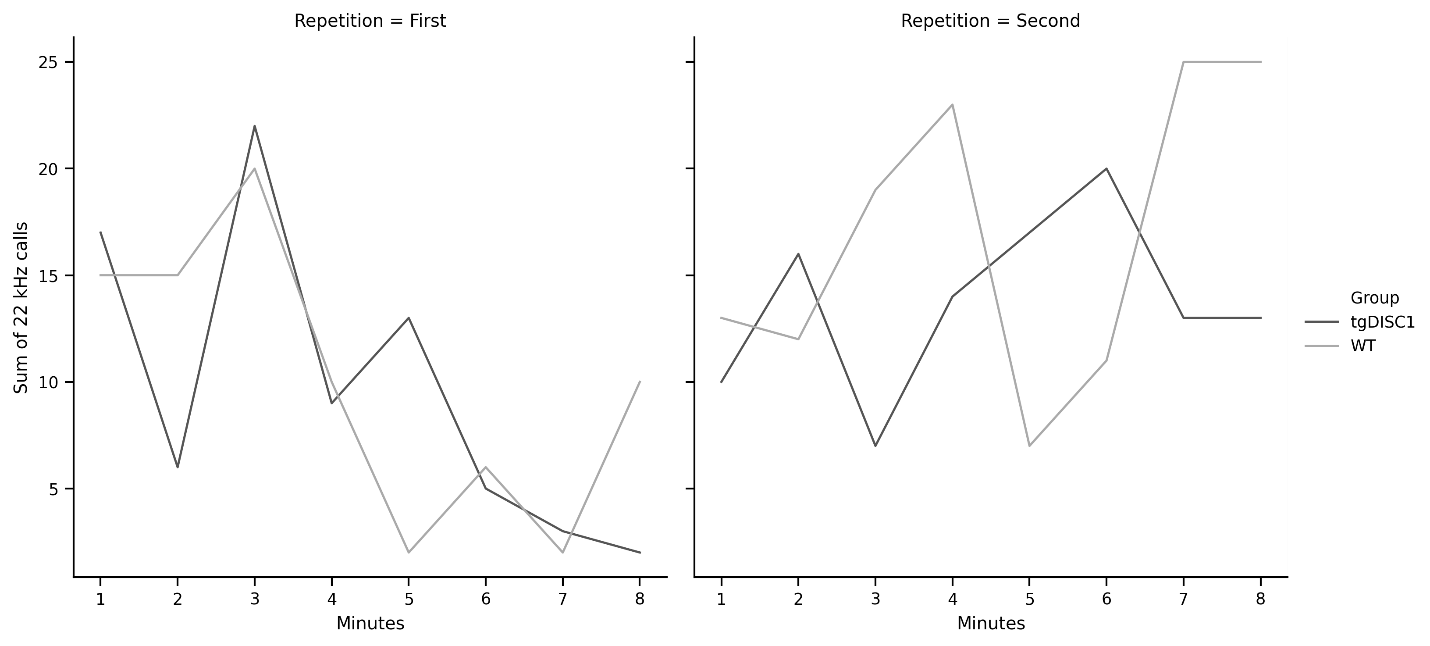


**Figure S3**. The sum of each group in vocalization of 22 kHz calls per minute across all conditions, per repetition.


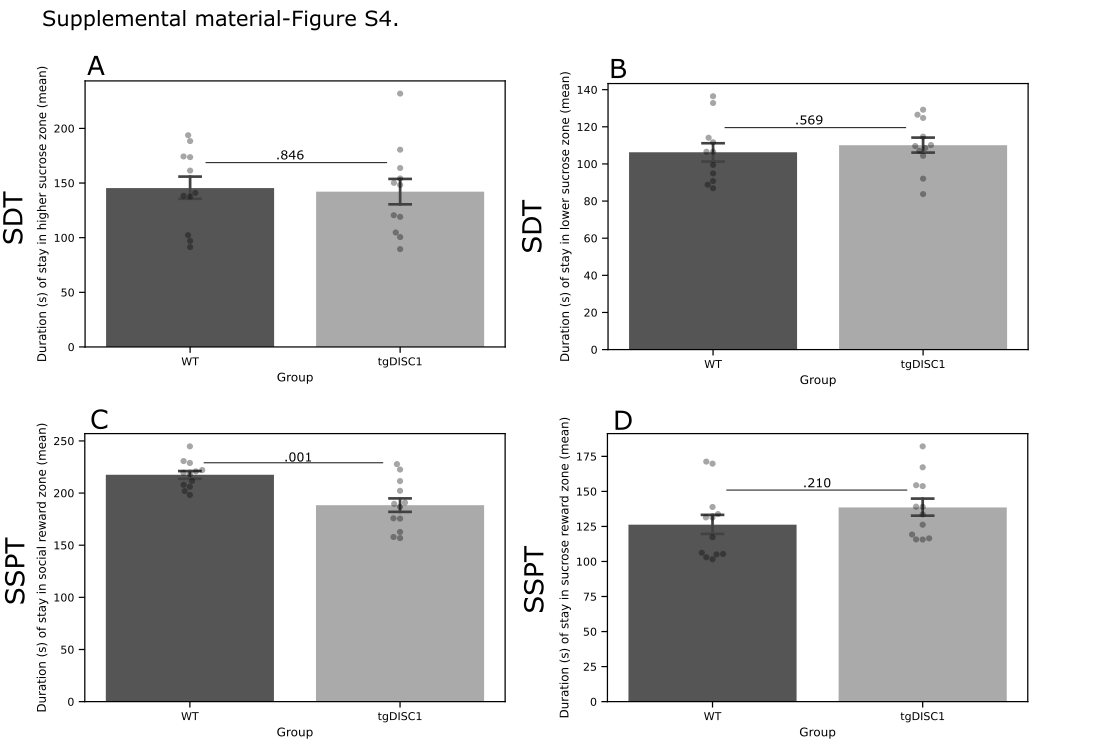


**Figure S4**. Mean of the duration of stay (s) per animal across all conditions and repetitions for each task (SDT and SSPT) and zone ([SDT: Higher and lower sucrose zone, SSPT: Social and Sucrose reward zone]). **A** between groups’ difference in duration (s) of stay in the higher sucrose zone. **B** between groups’ difference in duration (s) of stay in the lowers sucrose zone. **C** between groups’ difference in duration (s) of stay in the social reward zone. **D.** between groups’ difference in duration (s) of stay in the sucrose reward zone.


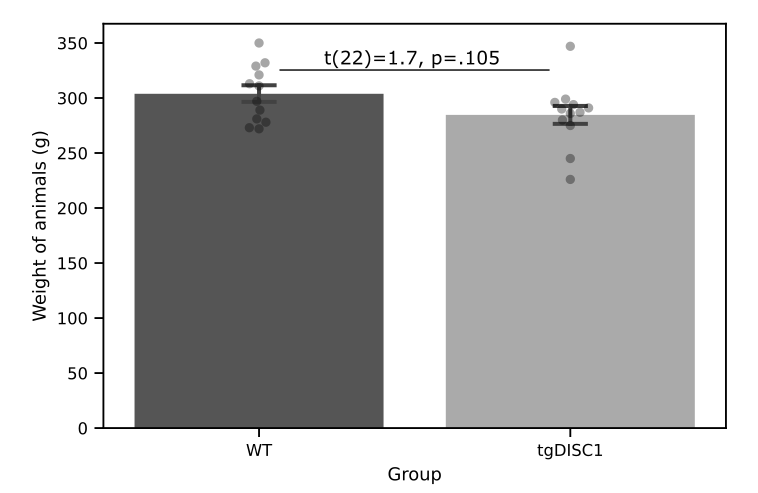


**Figure S5.** The comparison of the weight of the group members at the start of the experiment.

|  | | | | | |
| --- | --- | --- | --- | --- | --- |
|  | | | | | |
| **Table S1.** The one-sample t-tests show the significant preference of both groups for the higher sucrose in all three conditions of the SDT phase. Except for the 2% vs. 5% condition, where tgDISC1 shows a borderline significant preference (this is only due to the strong preference of one animal of the tgDISC1 group for the lower sucrose, see Fig 2. B), both groups significantly preferred the higher sucrose in the second replicate under other two conditions.  **One-Sample Test** | | | | | |
| Group | | Test Value = 50 | | | |
|  |  | t | df | Sig. (2-tailed) | Mean Difference |
|  |  |  |  |  |  |
| tgDISC1 | higher_sucrose_2vs.5 | 1.8 | 11 | .098 | 7.5 |
|  | higher_sucrose_2vs.10 | 10.8 | 11 | .000 | 23.8 |
|  | higher_sucrose_5vs.10 | 4.0 | 11 | .002 | 18.3 |
| WT | higher_sucrose_2vs.5 | 3.1 | 11 | .010 | 10.3 |
|  | higher_sucrose_2vs.10 | 3.3 | 11 | .006 | 15.8 |
|  | higher_sucrose_5vs.10 | 5.9 | 11 | .000 | 17.0 |

| **One-Sample Test** | | | |
| --- | --- | --- | --- |
| Group | | Test Value = 50 | |
|  |  | 95% Confidence Interval of the Difference | |
|  |  | Lower | Upper |
| tgDISC1 | higher_sucrose_2vs. 5 | -1.6 | 16.7 |
|  | higher_sucrose_2vs.10 | 18.9 | 28.6 |
|  | higher_sucrose_5vs.10 | 8.3 | 28.3 |
| WT | higher_sucrose_2vs. 5 | 3.0 | 17.6 |
|  | higher_sucrose_2vs.10 | 5.4 | 26.3 |
|  | higher_sucrose_5vs.10 | 10.7 | 23.4 |

**Table S2.** The result of one-sample t-tests on the preference of the social reward versus the three concentrations of sucrose, across conditions, both repetitions. In all conditions, both groups significantly preferred the social reward.

| **One-Sample Test** | | | | | |
| --- | --- | --- | --- | --- | --- |
| Group | | Test Value = 50 | | | |
|  |  | t | df | Sig. (2-tailed) | Mean Difference |
|  |  |  |  |  |  |
| tgDISC1 | social_reward_vs_2 | 4.0 | 11 | .002 | 8.9 |
|  | social_reward_vs_5 | 3.4 | 11 | .005 | 7.8 |
|  | social_reward_vs_10 | 3.4 | 11 | .005 | 5.9 |
| WT | social_reward_vs_2 | 12.4 | 11 | .000 | 16.2 |
|  | social_reward_vs_5 | 8.9 | 11 | .000 | 16.0 |
|  | social_reward_vs_10 | 2.2 | 11 | .046 | 8.7 |

| **One-Sample Test** | | | |
| --- | --- | --- | --- |
| Group | | Test Value = 50 | |
|  |  | 95% Confidence Interval of the Difference | |
|  |  | Lower | Upper |
| tgDISC1 | social_reward_vs_2 | 4.1 | 13.8 |
|  | social_reward_vs_5 | 2.8 | 12.7 |
|  | social_reward_vs_10 | 2.1 | 9.7 |
| WT | social_reward_vs_2 | 13.3 | 19.0 |
|  | social_reward_vs_5 | 12.1 | 20.0 |
|  | social_reward_vs_10 | .20 | 17.2 |

**References.**

1. Demaestri, C., Brenhouse, H. C. & Honeycutt, J. A. 22 kHz and 55 kHz ultrasonic vocalizations di ff erentially in fl uence neural and behavioral outcomes : Implications for modeling anxiety via auditory stimuli in the rat. **360**, 134–145 (2019).

2. Litvin, Y., Blanchard, D. C. & Blanchard, R. J. Rat 22 kHz ultrasonic vocalizations as alarm cries. *Behavioural Brain Research* vol. 182 166–172 (2007).

3. Jelen, P., Soltysik, S. & Zagrodzka, J. 22-kHz Ultrasonic v ocalization in rats as an index of anxiety but not fear : beha v ioral and pharmacological modulation of affecti v e state. **141**, 63–72 (2003).
